# Frequent nocturnal awakening in children: prevalence, risk factors, and associations with subjective sleep perception and daytime sleepiness

**DOI:** 10.1186/1471-244X-14-204

**Published:** 2014-07-30

**Authors:** Liwen Li, Jiwei Ren, Lei Shi, Xinming Jin, Chonghuai Yan, Fan Jiang, Xiaoming Shen, Shenghui Li

**Affiliations:** MOE - Shanghai Key Laboratory of Children’s Environmental Health, Xinhua Hospital, School of Medicine, Shanghai Jiao Tong University, Kong Jiang Road No.1665, Shanghai, 200092 China; School of Public Health, Shanghai Jiao Tong University, South Chongqing Road No.227, Shanghai, 200027 China; Shanxi Third People’s Hospital, Shanxi Cancer research center, Zhigong New Street No.3, Taiyuan, 030013 Shanxi, China; Shanghai Children’s Medical Center, School of Medicine, Shanghai Jiao Tong University, Dongfang Road No.1678, 2000127 Shanghai, China

**Keywords:** Children, Nocturnal awakening, Prevalence, Predisposing factors, Daytime sleepiness

## Abstract

**Background:**

Nocturnal awakening is the most frequent insomnia complaint in the general population. In contrast to a growing knowledge based on adults, little is known about its prevalence, correlated factors, and associations with subjective sleep perception and daytime sleepiness in children. This study was designed to assess the prevalence and the correlate factors of frequent nocturnal awakening (FNA) among Chinese school-aged children. Furthermore, the associations of FNA with subjective sleep perception and daytime sleepiness were examined.

**Methods:**

A random sample of 20,505 children aged 5.00 to 11.92 years old (boys: 49.5% vs. girls: 50.5%) participated in a cross-sectional survey, which was conducted in eight cities of China. Parent-administered questionnaires were used to collect information on children’s sleep behaviors, sleep perception, and potential influential factors of FNA from six domains. Univariate and multivariate logistic regression models were performed.

**Results:**

The prevalence of FNA was 9.8% (10.0% for boys vs. 8.9% for girls) in our sampled children. The prominent FNA-related factors inclued biological health problems, such as overweight/obesity (OR = 1.70), chronic pain during night (OR = 2.47), and chronic respiratory condition (OR = 1.23), poor psychosocial condition, such as poor mental and emotional functioning (OR = 1.34), poor sleep hygiene, such as frequently doing exciting activities before bedtime (OR = 1.24) and bedtime resistance (OR = 1.42), and parents’ history of insomnia (OR = 1.31). FNA was associated with subjective poor sleep quality (OR = 1.24), subjective insufficient sleep (OR = 1.21), and daytime sleepiness (OR = 1.35).

**Conclusion:**

FNA was associated with poor sleep and daytime sleepiness. Compared to sleep environment and family susceptibility, chronic health problems, poor psychosocial condition, and poor sleep hygiene had greater impact on FNA, indicating childhood FNA could be partly prevented by health promotion, by psychological intervention, and by improving sleep hygiene routine.

**Electronic supplementary material:**

The online version of this article (doi:10.1186/1471-244X-14-204) contains supplementary material, which is available to authorized users.

## Background

Insomnia, as an public health concern, has been an important topic in sleep and psychology research fields. Meanwhile, more attention is being given to nocturnal awakening, one of the most frequent insomnia complaints in the general population [1, 2]. Epidemiological studies suggested that the prevalence of nocturnal awakening, depending on the criteria used, ranged from 9.4%-23.8% in adolescents [3–5], 18.0%-35.0% in the general adults, and reached up to 65.0% among older individuals [6–8]. Accumulating studies demonstrated that the disruption and fragmentation of sleep were associated with a wide range of neurobehavioral and psychological impairments, including poor concentration, excessive daytime sleepiness and chronic fatigue, and predisposition to psychiatric morbidities [2, 7, 9, 10]. Moreover, recent studies further revealed that insomnia may increase the risk of hypertension, cardiovascular symptoms, and type 2 diabetes [11–13].

Although the exact etiology and pathophysiology of insomnia are still far from clear, a number of risk factors have been identified to be associated with the development of insomnia, such as age, gender, socio-economic status, sleep hygiene, sleep environment, stressful life events, and psychiatric and medical conditions [1, 2, 6, 7, 12–14]. In addition, studies both in adults and children found that insomnia symptoms had the phenomenon of familial aggregation [15–17]. Family history was related to the earlier onset of insomnia symptoms in the probands [15–17], which emphasized the family susceptibility of insomnia. Taken together, the interactions and co-existing of genetic factors, biological condition, and environments could play a pivotal role in the intrigue of insomnia symptoms [1, 18].

Although less well characterized than in adolescents and adults, insomnia symptoms in children are common. The prevalence of nocturnal awakening in school-aged children have been reported to be ranged as 4.0%-13.6% [17, 19–22]. It was demonstated that childhood insomnia symptom could result in a impairment of daytime functioning not only in child but also in family [5, 17, 20, 23]. A longitudinal study confirmed that approximately 60% of childhood insomnia symptoms, including nocturnal awakening, can enter into early adolescents [19]. Meanwhile, it was demonstrated that the disruption and fragmentation of sleep during childhood and adolescence were independent predictors for a number of risky behaviors, such as substance abuse, impulsive attack, and even delinquency, in later life [5, 24–27].

While the importance is becoming quite impressive, childhood insomnia symptoms are critically less well-studied and poorly understood. Therefore, the present epidemiological study was designed to investigate the prevalence of frequent nocturnal awakening (FNA), one of the core symptoms of insomnia, to examine the associations of FNA with multidimensional factors, including sociodemographic characteristics, biological chronic health problems, psychosocial conditions, sleep environments, sleep hygiene, and family history of insomnia, and to assess the impact of FNA on subjective sleep quality/quantity and daytime sleepiness among a large nationally representative sample of school-aged children in Mainland, China.

## Methods

### Study design and subjects

The recruitment strategy and procedure of the study were described elsewhere [28, 29]. In short, based on a cross-sectional design, 55 elementary schools from eight cities were sampled, using a cluster-stratified selection procedure. Students who were eligible to participate in this study were invited to take a questionnaire on sleep behaviors and personal and family information to their parents, with a cover letter explaining the objectives of the project and instructions on how to complete the questionnaire. Parents were told that the participation in the survey was voluntary and informed consent was signed. Of 23,791 children recruited from six grades of the chosen schools, 22,018 (92.5%) returned completed questionnaires.

To eliminate the possible pubertal influences on the results of our study, children who had entered pubertal development were considered to be excluded. In China, adolescents refer to children aged 12/13 to 17/18 years old [30, 31]. Therefore, 1313 children ≥ 12.00 years were excluded from the sample. In addition, 200 (0.96%) children were excluded from further analyses because of missing information on frequency of awakening during night. The final sample consisted of 20,505 children (49.5% boys vs. 50.5% girls). The mean age was 8.98 years (SD = 1.59 years, ranged from 5.00 to 11.92 years).

The ethical application and consent procedure of this study were approved by the Ministry of Education of the People’s Republic of China and Ethics Committee of Shanghai Jiaotong University School of Medicine.

### Measures

The Children’s Sleep Habits Questionnaire (CSHQ), a parents-administrated questionnaire, was used to assess children’s sleep behaviors [32]. The 33 CSHQ items were conceptually grouped into 8 subscales: bedtime resistance, sleep onset delay, irregular sleep duration, sleep anxiety, night awakening, parasomnias, sleep-disordered breathing, and daytime sleepiness [32]. A Chinese version of the CSHQ (as shown in Additional file 1: Table S1) was developed by translation and back translation and has been used previously with proven excellent sensitivity and reliability [29].

In the present study, FNA was defined as: i. Nocturnal awakenings occurred at least two times per night for at least two nights per week; or/and ii. Nocturnal awakenings occurred one time per night for at least two nights per week accompanied with difficulty in resuming sleep (awakening duration ≥ 30minutes).

There were two items (“How often does your child’s sleep quality is good?” and “How often does your child’s sleep duration is insufficient?”) regarding children’s subjective sleep quality and sleep quantity in the CSHQ. Based on the response, sleep quality was recode into two categories: good (good sleep ≥ 5 nights per week) and poor (good sleep < 5 nights per week). Similarly, sleep quantity was recode into two categories: sufficient (insufficient sleep < 2 nights per week) and insufficient (insufficient sleep ≥ 2 nights per week).

Daytime sleepiness subscale included eight items related to awakening difficulties and daytime tiredness. The subscale score was calculated with the higher the score, the higher level the sleepiness. Based on the distribution of subscale score, daytime sleepiness was defined as subscale score ≥ 75^th^ percentile.

The Children’s Psychosocial Screening Scale (CPSS, as shown in Additional file 2: Table S2) was designed specifically to assess children’s psychosocial functioning for this study based on literature review, qualitative research, and a pilot study. The CPSS includes 14 items and the 14 items are conceptually grouped into 3 domains: mental and emotional condition (5 items), peer acceptance and social function (5 items), and parenthood (4 items). For each item of CPSS, the response was assessed on a 5-point Likert scale (1 = rarely, 5 = always). The total score for each domain was then calculated with the higher the score, the better well-being.

The sensitivity and reliability of the CPSS was assessed and proved to be excellent (Cronbach’s alpha’s for the internal consistency were 0.89 for the overall questionnaire and ranged from 0.88 to 0.91 for subscales; Intraclass correlation coefficients for the test-retest reliability were 0.85 for the overall questionnaire and ranged from 0.60 to 0.88 for subscales).

To analyzing the impact of psychosocial functioning on nocturnal awakening, the each domain score was recode into two categories: “0” for the score ≥ 25^th^ and “1” for the score < 25^th^. In the present study, domain score < 25^th^ was defined as poor psychosocial functioning.

The twenty-four possible risk factors regarding frequent nocturnal awakening were conceptually grouped into six dimensions: sociodemographic characteristics, biological chronic health problems, psychosocial conditions, sleep environments, sleep hygiene, and family history of insomnia.

Sociodemographic variables: gender, age, ethnicity, family structure (single parent family, large family [the family with family members of grandparents, parents, and child], and nuclear family), parents’ educational levels (illiteracy, elementary or middle school, high school, college or university, and above university), and household income (<800, 800–2500, and ≥2500 Renminbi [RMB][yuan]/person/month).

Biological chronic health problem variables: overweight/obesity status (yes/no, overweight/obesity was defined by the standardized internationally referenced gender- and age-specific BMI [weight in kg/height in m^2^] cut-offs) [33], chronic pain during night (yes/no, with definition of having minor of moderate pain in head, stomach, or limbs during night ≥ 2 nights per week in recent four weeks), ADHD (yes/no, with definition of being ever diagnosed with ADHD by pediatricians), chronic respiratory condition (yes/no, with definition of being ever diagnosed with allergic rhinitis and bronchitis, asthma, or tonsil/adenoidal hypertrophy by pediatricians), and chronic food or drug allergy (yes/no).

Psychosocial condition variables: mental and emotional condition (good/poor), peer acceptance and social function (good/poor), and parenthood (good/poor).

Sleep environment variables: sleep arrangements (routine bed-sharing, routine room-sharing, and routine sleeping alone or other; routine bed- and room-sharing was defined as sharing a bed or room with parents/caregivers 5–7 nights per week), bedroom intrusive light during night (yes/no), and bedroom intrusive noise during night (yes/no).

Bedtime hygiene variables: drinks with caffeine use after 6:00pm [(Parents were asked to report on a four-point scale (usually, often, and occasional/no) and then the responses were dichotomized into a binary variable in the final analyses], doing exciting activities (for example, playing outside, playing video/internet games, and watching violent/stimulating television programs) during bedtime [(Parents were asked to report on a four-point scale (usually, often, and occasional/no) and then the responses were dichotomized into a binary variable in the final analyses], bedtime resistance (having trouble settling down or need help getting to sleep, for example, need to listen to music, watch television, take medicine, or need parents/caregivers in the bed) (yes/no), and irregular bedtime (yes/no).

Family history of insomnia: grandparents’ history of insomnia (yes/no) and parents’ history of insomnia (yes/no).

### Statistical analysis

Statistical descriptions were made by use of the mean, standard deviation for continuous variables, and percentage for categorical variables.

To identify risk factors regarding FNA, the logistic regression analyses were performed, with “1” for FNA and “0” for non- FNA. Unadjusted odds ratios (OR) and 95% confidence intervals (CI) were calculated using univariate logistic regression (Table 1). Adjustments were further made by the multivariate regression models following a four-step procedure. Each model included additional variables to assess increasingly proximate determinants of FNA. Firstly, a simple model (model I) adjusted only for sociodemographic variables (Tables 2 and 3). Secondly, variables regarding biological health problems and psychosocial conditions (Table 2) or sleep environments (Table 3) were further included (model II). Thirdly, variables regarding family history of insomnia (Table 2) or sleep hygiene (Table 3) were additionally further included (model III). Finally, a full model (model IV) was established by adjusting all factors regarding sociodemographic characteristics, biological and psychosocial factors, and sleep environments, sleep hygiene, and family history simultaneously.

**Table 1:** **Associated factors regarding nocturnal awakening by univariate logistical regression models (N = 20 505)**

Variables (n, %)	Prevalence of nocturnal awakening n (%)	Univariate regression models OR (95% CI)	***P***value
**Demographic characteristics**			
Age (years)			<.001
5-6 (2534, 12.4%)	196 (7.7)	1.00	
7- (3781, 18.6%)	343 (9.1)	1.19 (0.99-1.43)	.063
8- (3817, 18.7%)	351 (9.2)	1.18 (0.98-1.42)	.074
9- (3781, 18.2%)	377 (10.0)	1.32 (1.10-1.58)	.002
10- (3703, 17.1%)	370 (10.0)	1.32 (1.10-1.58)	003
11- (2762, 13.6%)	298 (10.9)	1.44 (1.19-1.74)	<.001
Gender (%)			
Boys (10095, 49.5%)	1005 (10.0)	1.13 (1.03-1.24)	.013
Girls (10306, 50.5%)	921 (8.9)	1.00	
Ethnicity			
Han ethnic group (19346, 94.9%)	1809 (9.4)	1.00	
Minority ethnic group (1038, 5.1%)	114 (11.0)	1.20 (0.98-1.46)	.080
**Socioeconomic characteristics**			
Family income			<.001
<800 (3903, 19.3%)	567 (14.5)	2.57 (2.22-2.98)	<.001
800-2500 (11460, 56.6%)	1033 (9.0)	1.50 (1.31-1.71)	<.001
≥2500 (4902, 24.2%)	304 (6.2)	1.00	
Family structure			<.001
Single parent family (1081, 5.3%)	150 (13.9)	1.53 (1.27-1.83)	<.001
Large family (6478, 31.7%)	551 (8.5)	0.88 (0.79-0.98)	.019
Nuclear family (12852, 63.0%)	1226 (9.5)	1.00	
Mather’s education level			<.001
Low (5687, 28.2%)	853 (15.0)	2.91 (2.58-3.28)	<.001
Medium (6716, 33.3%)	589 (8.8)	1.58 (1.39-1.80)	<.001
High (7741, 38.4%)	443 (5.7)	1.00	
Father’s education level			<.001
Low (4884, 24.0%)	764 (15.6)	2.86 (2.54-3.22)	<.001
Medium (6969, 34.2%)	639 (9.2)	1.56 (1.38-1.76)	<.001
High (8538, 41.9%)	520 (6.1)	1.00	
**Biological chronic health problems**			
Overweight/obesity			
Yes (4119, 20.6%)	660 (16.2)	1.87 (1.42-2.48)	<.001
No (15878, 79.4%)	1471 (9.3)	1.00	
Chronic pain during night			
Yes (1362, 6.7%)	299 (22.0)	3.03 (2.64-3.47)	<.001
No (18995, 93.3%)	1616 (8.5)	1.00	
History of ADHD diagnosis			
Yes (921, 4.5%)	151 (16.4)	1.95 (1.63-2.34)	<.001
No (19568, 95.5%)	1787 (9.1)	1.00	
Chronic respiratory condition			
Yes (7000, 34.2%)	754 (10.8)	1.26 (1.14-1.39)	<.001
No (13457, 65.8%)	1178 (8.8)	1.00	
Food/drug allergy			
Yes (1160, 5.7%)	122 (10.5)	1.13 (0.93-1.38)	.046
No (19331, 94.3%)	1816 (9.4)	1.00	
**Psychosocial condition**			
Poor mental and emotional condition			
Yes (6856, 33.4%)	844 (12.3)	1.61 (1.46-1.77)	<.001
No (13649, 66.6%)	1097 (8.0)	1.00	
Poor peer acceptance/social functioning			
Yes (5685, 27.7%)	663 (11.7)	1.40 (1.27-1.55)	<.001
No (14820, 72.3%)	1278 (8.6)	1.00	
Poor parenthood			
Yes (6495, 31.7%)	762 (11.7)	1.45 (1.31-1.59)	<.001
No (14010, 68.35%)	1179 (8.4)	1.00	
**Sleep environments**			
Sleep arrangements			.215
Bed-sharing (4817, 23.5%)	477 (9.9)	1.09 (0.97-1.22)	.145
Room-sharing (3035, 14.8%)	302 (10.0)	1.09 (0.96-1.25)	.192
Sleeping alone (12653, 61.7%)	1162 (9.2)	1.00	
Bedroom intrusive noise			
Yes (861, 4.2%)	112 (13.0)	1.46 (1.19-1.79)	<.001
No (19577, 95.8%)	1821 (9.3)	1.00	
Bedroom intrusive light			
Yes (2173, 10.6%)	279 (13.0)	1.51 (1.32-1.72)	<.001
No (17880, 89.4%)	1660 (9.1)	1.00	
**Bedtime hygiene**			
Having drinks with caffeine after 6:00 pm			
Usually/often (7935, 38.8%)	892 (11.2)	1.39 (1.27-1.53)	<.001
Occasionally/never (12512, 61.2%)	1043 (8.3)	1.00	
Dong exciting activities before bedtime			
Usually/often (6092, 29.8%)	758 (12.4)	1.59 (1.44-1.75)	<.001
Occasionally/never (14331, 70.2%)	1179 (8.2)	1.00	
Bedtime resistance			
Yes (14421, 71.7%)	1528 (10.6)	1.65 (1.47-1.85)	<.001
No (5690, 28.3%)	382 (6.7)	1.00	
Irregular bedtime			
Yes (5952, 29.1%)	653 (11.0)	1.27 (1.15-1.40)	<.001
No (14518, 70.8%)	1285 (8.9)	1.00	
**Family history of insomnia**			
Grandparents’ history of insomnia			
Yes (1778, 8.7%)	179 (10.1)	1.08 (0.92-1.27)	.365
No (18727, 91.3%)	1762 (9.4)	1.00	
Parents’ history of insomnia			
Yes (3369, 16.4%)	455 (13.5)	1.64 (1.47-1.84)	<.001
No (17136, 83.6%)	1486 (8.7)	1.00	

Family income was expressed in RMB (yuan)/person/month.

OR: odds ratio; CI: confidence interval.

**Table 2:** **Biological chronic health problems, psychosocial condition, and family history regarding FNA by multivariate logistical regression models (N = 20,505)**

Variables	Model I		Model II		Model III		Model IV	
	Adjusted OR (95% CI)	***P***value	Adjusted OR (95% CI)	***P***value	Adjusted OR (95% CI)	***P***value	Adjusted OR (95% CI)	***P***value
**Biological health problems**								
Overweight/obesity vs. none	1.89 (1.40-2.55)	<.001	1.80 (1.32-2.44)	<.001	1.78 (1.31-2.42)	<.001	1.70 (1.25-2.35)	<.001
Chronic pain during night vs. none	2.87 (2.48-3.32)	<.001	2.67 (2.29-3.10)	<.001	2.60 (2.24-3.02)	<.001	2.47 (2.12-2.88)	<.001
History of ADHD diagnosis vs. none	1.56 (1.28-1.89)	<.001	1.29 (1.05-1.59)	.014	1.25 (1.02-1.54)	.033	1.20 (0.98-1.49)	.078
Chronic respiratory condition vs. none	1.42 (1.28-1.57)	<.001	1.27 (1.14-1.41)	<.001	1.25 (1.12-1.39)	<.001	1.23 (1.10-1.37)	<.001
Food/drug allergy vs. none	1.17 (0.95-1.44)	.147	0.98 (0.78-1.21)	.824	0.97 (0.78-1.20)	.759	0.99 (0.80-1.23)	.916
**Psychosocial condition**								
Poor mental and emotional condition		<.001		<.001		<.001		<.001
Yes vs. no	1.53 (1.41-1.71)		1.36 (1.25-1.53)		1.35 (1.21-1.53)		1.34 (1.20-1.49)	
Poor peer acceptance/social functioning		<.001		.107		.113		.110
Yes vs. no	1.22 (1.10-1.34)		1.07 (0.96-1.20)		1.06 (0.96-1.21)		1.06 (0.98-1.22)	
Poor parenthood		<.001		.008		.018		.063
Yes vs. no	1.35 (1.23-1.50)		1.16 (1.05-1.28)		1.13 (1.01-1.26)		1.12 (1.00-1.23)	
**Family history of insomnia**								
Grandparents’ history of insomnia		.227		.851		.705		.506
Yes vs. no	1.11 (0.94-1.32)		0.98 (0.82-1.18)		0.97 (0.81-1.56)		0.96 (0.82-1.14)	
Parents’ history of insomnia		<.001		<.001		<.001		<.001
Yes vs. no	1.52 (1.35-1.71)		1.34 (1.18-1.51)		1.34 (1.18-1.52)		1.31 (1.15-1.48)	

OR: odds ratio; CI: confidence interval.

Model I adjusted for demographic and socioeconomic characteristics;

Model II adjusted for demographic and socioeconomic characteristics, biological health problems, and psychosocial conditions;

Model III adjusted for demographic and socioeconomic characteristics, biological health problems, psychosocial conditions, and family history of insomnia;

Model IV adjusted for demographic and socioeconomic characteristics, biological health problems, psychosocial conditions, family history, sleep environments, and bedtime hygiene simultaneously.

**Table 3:** **Sleep environments and bedtime hygiene regarding FNA by multivariate logistical regression models (N = 20,505)**

Variables	Model I		Model II		Model III		Model IV	
	Adjusted OR (95% CI)	***P***value	Adjusted OR (95% CI)	***P***value	Adjusted OR (95% CI)	***P***value	Adjusted OR (95% CI)	***P***value
**Sleep environments**								
Sleep arrangements		.118		.141		.859		.741
Bed-sharing vs. sleeping alone	1.14 (1.01-1.29)	.039	1.13 (1.00-1.28)	.050	0.99 (0.88-1.13)	.927	0.97 (0.86-1.10)	.548
Room-sharing vs. sleeping alone	1.05 (0.92-1.22)	.467	1.06 (0.92-1.22)	.424	1.04 (0.90-1.20)	.620	1.04 (0.90-1.21)	.514
Bedroom intrusive noise vs. none	1.13 (0.91-1.40)	.286	1.12 (0.90-1.39)	.326	1.09 (0.87-1.36)	.462	1.01 (0.81-1.27)	.9207
Bedroom intrusive light vs. none	1.23 (1.06-1.42)	.006	1.22 (1.05-1.41)	.009	1.18 (1.01-1.37)	.032	1.15 (1.00-1.34)	.069
**Bedtime hygiene**								
Having caffeine drinks after 6:00pm		<.001		<.001		.002		.054
Usually/often vs. occasionally/no	1.28 (1.16-1.41)		1.27 (1.15-1.40)		1.18 (1.07-1.30)		1.12 (0.99-1.24)	
Doing exciting activities before bedtime		<.001		<.001		<.001		<.001
Usually/often vs. occasionally/no	1.43 (1.29-1.58)		1.41 (1.27-1.56)		1.31 (1.18-1.46)		1.24 (1.10-1.39)	
Bedtime resistance vs. none	1.59 (1.41-1.80)	<.001	1.57 (1.39-1.78)	<.001	1.57 (1.37-1.80)	<.001	1.42 (1.24-1.63)	<.001
Irregular bedtime vs. none	1.12 (1.01-1.24)	.041	1.11 (1.00-1.23)	.057	0.92 (0.82-1.03)	.154	0.91 (0.81-1.02)	.075

OR: odds ratio; CI: confidence interval;

Model I adjusted for demographic and socioeconomic characteristics;

Model II adjusted for demographic and socioeconomic characteristics and sleep environments;

Model III adjusted for demographic and socioeconomic characteristics, sleep environments, and bedtime hygiene;

Model IV adjusted for demographic and socioeconomic characteristics, biological health problems, psychosocial conditions, family history, sleep environments, and bedtime hygiene simultaneously.

To assess the impact of FNA on subjective sleep quality, subjective sleep quantity, and daytime sleepiness, the multivariate regression models were conducted by a two-step procedure. Model I adjusted only for sociodemographic variables and model II further controlled for bedtime, wake time, sleep duration, and all other sleep problems in CSHQ (bedtime resistance, sleep onset delay, irregular sleep duration, sleep anxiety, parasomnias, and sleep-disordered breathing).

All analyses were performed using the Statistical Package for Social Sciences (SPSS) for Windows, version 13.5 (SPSS Inc, Chicago, IL, USA). In the presentation of the results, the statistical significance was set at *P* value < 0.05 (two tailed).

## Results

### Prevalence of FNA

Our survey showed that the prevalence of FNA in our sampled children was 9.8%. Significantly gender difference was found (10.0% for boys vs. 8.9% for girls; χ^2^ = 6.19, *P* = 0.007). In addition, the prevalence of FNA increased with increasing age (χ^2^ = 59.97, *P* < 0.001).

### Predisposing factors of FNA by logistical analyses

The unadjusted OR with 95% CI for associatons between FNA and the possible influential actors are demonstrated in Table 1. It can be seen that, except for ethnicity, sleep arrangements, and food/drug allergy, all other factors were significantly associated with FNA in the univariate regression models.

The associations of sociodemographic factors with FNA are shown in Additional file 3: Table S3. After controlling for all covariates, four factors (older age, lower family income, and lower level of parents’ educational levels) were significantly associated with an increased likelihood of FNA. Meanwhile, large family was a protective factor for FNA (Model IV).

The associations between FNA and biological health problems, psychosocial condition, and family history of insomnia are shown in Table 2. After adjusting only for sociodemographic characteristics, those biological health problems, such as overweight/obesity, chronic pain during night, history of ADHD diagnosis, and chronic respiratory condition, all poor psychosocial condition, and parents’ history of insomnia were significantly associated with an increased likelihood of FNA (Model I). After additionally adjusting for the all bio-psychosocial factors and family history of insomnia, seven factors, such as overweight/obesity, chronic pain during night, history of ADHD diagnosis, chronic respiratory condition, poor mental and emotional functioning, poor parenthood, and parents’ history of insomnia remained statistically significant (Model III). After controlling for sleep environments and sleep hygiene simultaneously, except for history of ADHD diagnosis and poor parenthood, all other five factors remained to be independent predictors for FNA (Model IV).

The associations between FNA and sleep environments and sleep hygiene are shown in Table 3. After adjusting only for demographic and socioeconomic characteristics, six factors (bed-sharing, bedroom intrusive light, frequently having drinks with caffeine, frequently doing exciting activities before bedtime, bedtime resistance, and irregular sleep habits) were significantly associated with an increased likelihood of nocturnal awakening (Model I). After additionally controlling for sleep environments and sleep hygiene, four factors remained statistically significant (Model III). After further adjusting for all bio-psychosocial factors and family history of insomnia simultaneously, two factors, frequently doing exciting activities before bedtime and bedtime resistance, remained to be independent related to FNA (Model IV).

### The impacts of FNA on subject sleep quality/quantity and daytime sleepiness

After controlling for sociodemographic characteristics, sleep problems (bedtime resistance, irregular sleep habits, sleep anxiety, parasomnia, and sleep disordered breathing), sleep/wake habits, and sleep duration, FNA was found to be an independent risk factor for subjective poor sleep quality (OR:1.24, *P* < 0.001), subjective insufficient sleep (OR:1.21, *P* < 0.001), and daytime sleepiness (OR:1.35, *P* < 0.001) (Table 4, Model II).

**Table 4:** **The impact of FNA on subject sleep quality/amount and daytime sleepiness (N = 20,505)**

	Model I		Model II	
	Adjusted OR (95% CI)	***P***value	Adjusted OR (95% CI)	***P***value
Nocturnal awakening and subject sleep quality (poor vs. good)				
Nocturnal awakening	1.46 (1.32-1.61)	<.001	1.24 (1.10-1.40)	<.001
None	1.00		1.00	
Nocturnal awakening and subject sleep amount (insufficiency vs. sufficiency)				
Nocturnal awakening	1.48 (1.34-1.63)	<.001	1.21 (1.06-1.41)	.007
None	1.00		1.00	
Nocturnal awakening and daytime sleepiness (daytime sleepiness vs. none)				
Nocturnal awakening	1.64 (1.48-1.82)	<.001	1.35 (1.21-1.53)	<.001
None	1.00		1.00	

OR: odds ratio; CI: confidence interval;

Model I adjusted for demographic and socioeconomic characteristics;

Model II additionally adjusted for bedtime resistance, irregular sleep habits, sleep anxiety, parasomnia, sleep disordered breathing, bedtime, wake time, and sleep duration.

## Discussion

To the best of our knowledge, this was the largest epidemiological study on childhood FNA (n = 20,505). Our findings demonstrated that the prevalence of FNA was 9.8% (10.0% for boys vs. 8.9% for girls) in Chinese school-age children. Compared to sleep environment and family history, chronic health problems, poor psychosocial condition, and poor sleep hygiene had greater impact on FNA, indicating childhood FNA could be partly prevented by health promotion, by psychological intervention, and by improving the sleep hygiene routine. The findings should have important clinical and preventive implications for childhood sleep health promotion.

Based on limited studies, the prevalence of FNA in school-aged children was previously reported to range from 4.0% to 13.6%, depending on different frequency criteria [17, 19–22]. Our study indicated that 9.8% of Chinese school-aged children have FNA in recent four weeks, which was somewhat higher prevalent than most previously reported [17, 19–21]. Boys had higher prevalence of FNA among our sampled school-aged children. The possible explanation is that boys, compared to girl, were tended to have poorer sleep hygiene and have more biological health problems, such as overweight/obesity, ADHD, and food/drug allergy. By contrast, most studies in adolescents and adults demonstrated that females are more vulnerable to insomnia symptoms. [1, 2, 11] A study specifically examined the gender difference in insomnia, indicated that gender difference in insomnia maybe occur only after puberty [4]. In additional, our study found an age-dependent increasing tendency in the prevalence of FNA in our studied age group. The increasing prevalence of insomnia symptoms in this age group has previously been reported and may be explained [17, 19], or at least partly, by the age-dependent biological and psychosocial development in childhood [20, 21]. More studies are needed to shed light on the gender and age difference in insomnia, which could be valuable in interpreting the bio-psychosocial etiology of insomnia.

To the best of our knowledge, this was the first study to report associated factors regarding FNA from multidimensional perspectives in school-aged children. Our study included twenty-four potential influential factors elicited from six domains: sociodemographic characteristics, biological chronic health problems, psychosocial fitness, sleep environments, bedtime hygiene, and family history of insomnia.

Taking the potential confounding effects into account, the multivariate regression model demonstrated that eleven factors were associated with the FNA in our sampled children. The eleven factors covered six domains: demographic characteristics (older age), family structure and socioeconomic status (lower family income, lower level of parents’ educational levels), biological chronic health problems (overweight/obesity, chronic pain during night, and chronic respiratory conditions), psychosocial condition (poor mental and emotional functioning), bedtime hygiene (frequently doing exciting activities before bedtime and bedtime resistance), and family history of insomnia (parents’ history of insomnia). Among all these identified factors, six factors from three domains (biological chronic health problems, psychosocial condition, and bedtime hygiene) should be paid more close attention since these factors can be the important targets for clinical or preventive intervention.

Accumulating studies suggested that chronic health problems had negative impact on sleep maintaining [34–36]. Consistent with these studies, our results demonstrated that chronic pain during night and chronic respiratory conditions, such as allergic rhinitis and bronchitis, asthma, and tonsil/adenoidal hypertrophy, were associated with FNA in school-aged children. In additional, our study demonstrated that overweight/obesity was related to childhood FNA. A very recent study in adults got very similar findings that obesity was an independent risk factor for insomnia and daytime sleepiness [37]. Another study in adults demonstrated that people with nightly nocturnal awakening were easy to become overweight or obese [6]. Taken these findings together, it seems that there is a bidirectional regulation association between sleep and obesity. It has been known that sleep is a major modulator of hormonal release and glucose regulation [38]. Therefore, the association between overweight/obesity and insomnia is an interesting topic and deserve further exploring.

Based on a tripartite conceptual framework, which is widely used to explain the development of insomnia, psychological and emotional factors are involved in all stages during the course of insomnia [1, 20]. A longitudinal study specifically observed the course of insomnia and associations with stressful life events, psychological status, and health condition [35]. It was reported that psychological variables can play predisposing role in the onset and development of insomnia [35]. Although the association between psychological functioning and insomnia was well established in adults [1, 2, 7, 9–11], litter is known in childhood. Only one study in children aged 6 to 13 years reported that frequent temper outburst was a risk factor for childhood insomnia symptoms. [17] Our study demonstrated that poor mental and emotional fitness was associated with an increased FNA in Chinese school-aged children. The finding provided new information for understanding the relationship between psychological functioning and childhood insomnia.

Bedtime hygiene is usually defined as behavioral practices before bedtime, which have been proved to be associated with sleep latency, sleep quality, and sleep maintaining [39]. There was a general understanding of the importance of good sleep hygiene practice, such as avoiding alcohol, tobacco, and caffeine use before bedtime, following a regular bedtime routine; avoiding bedtime activities that are emotionally activating; and maintaining a stable sleep schedule, for high quality of sleep [40, 41]. Our study supported previous findings that poor sleep hygiene practice (e.g., bedtime struggling behaviors and doing exciting activities before bedtime) were strong predictors for interrupted sleep [40, 41].

Findings from this study revealed that FNA was an independent risk factor for subjective poor sleep and daytime sleepiness in children, supporting the recognition that poor sleep perception and daytime sleepiness were outcomes frequently encountered by individuals with insomnia symptoms. As well documented, daytime sleepiness was linked to several severe consequences, such as unstable and unreliable alertness and vigilance, poor cognitive capabilities, increased risk of making errors, and an increased risk of accidents [42–46]. Additional research is needed to elaborate the full spectrum of the psychological, neurobiological, and developmental consequences of childhood insomnia.

There are several limitations that should be considered in interpreting the results. The first limitation is the reliance on parental reported data on children’s sleep behaviors without objective confirmation, which may increased the possibility of rater biases and inaccuracy. Secondly, we collected information on children’s chronic health problems by parent-reported questionnaire but not clinical measure - a vigorous method, however, unavailable for large samples. Thirdly, although the sensitivity and reliability of the CPSS was assessed and proved to be excellent, the validity was not examined. Finally, although we presented the results from a rich data-set of possible risk factors, other factors may be significantly associated with school-aged children’s FNA.

## Conclusions

This study provided information on the prevalence of FNA and associated risk factors in Chinese school-aged children. Our findings suggested that FNA was common in school-aged children and associated factors included not only socioeconomic and environmental factors, but also bio-psychosocial and family history. Upon the recognition that FNA has potential severe complications due to increased sleep fragmentation, these findings, although should be further confirmed by prospective studies, had important clinical implication for formulating intervention and treatment schemes. Due to a very large sample recruited from eight cities with geographical and socioeconomic diversity and a good response rate (92.5%), the results of this study entailed extended information for understanding childhood FNA and the correlates.

## Authors’ information

Liwen Li and Jiwei Ren are co-first authors.

## Electronic supplementary material

Additional file 1: Table S1: The Chinese Version of Child’s Sleep Habits Questionnaire. (DOC 118 KB)

Additional file 2: Table S2: The Children’s Psychosocial Screening Scale. (DOC 54 KB)

Additional file 3: Table S3: Sociodemographic factors regarding FNA by multivariate logistical regression models (N = 20,505). (DOC 66 KB)

## Abbreviations

FNA: Frequent nocturnal awakening
CSHQ: Children’s sleep habits questionnaire
CPSS: Children’s psychosocial screening scale
OR: Odds ratios
CI: Confidence interval.
