# Supplementary material for: Frequent nocturnal awakening in children: prevalence, risk factors, and associations with subjective sleep perception and daytime sleepiness
Source: BMC Psychiatry. 2014 Jul 30;14:204. doi: 10.1186/1471-244X-14-204 (PMC4261897; doi:10.1186/1471-244X-14-204)
Supplement: Supplementary file 1 — Additional file 1: Table S1: The Chinese Version of Child’s Sleep Habits Questionnaire. (DOC 118 KB) [file 12888_2013_1708_MOESM1_ESM.doc]

**Supplemental S1.The Chinese Version of Child's Sleep Habits Questionnaire**

| The following statements are about your child’s sleep habits and possible difficulties with sleep.Think about the past week in your child’life when answering these questions.If last week was unusual for a specific reason(such as your child had an ear infection and did not sleep well or the tv set was broken),choose the most recent typical week.Answer USUALLY if something occurs 5 or more times in a week ;answer SOMETIMES if it occurs 2-4 times in a week;answer RARELY if something occurs never or 1 time during a week.Also please indicate whether or not the sleep habit is a problem by circling “YES” ,”NO” ,or “Not Applicable(N/A)”. |
| --- |

**Bedtime：**

Child's bedtime：week nights：▁▁▁▁ weekends：▁▁▁▁

|  | Usually  5-7times/week | Sometimes  2-4times/week | Rare/no  0-1times/week |
| --- | --- | --- | --- |
| 1. Child goes to bed at the same time at night | □ | □ | □ |
| 2.Child falls asleep within 20 minutes after going to bed | □ | □ | □ |
| 3. Child falls asleep in own bed | □ | □ | □ |
| 4. Child falls asleep in sibling's bed | □ | □ | □ |
| 5. Child falls asleep with rocking or rhythmic movement | □ | □ | □ |
| 6.Child needs special object to fall asleep(doll, special blanket, etc) | □ | □ | □ |
| 7.Child needs parents in room to fall asleep | □ | □ | □ |
| 8. Child is ready to go to bed at bedtime | □ | □ | □ |
| 9. Child resists going to bed at bedtime | □ | □ | □ |
| 10.Child struggles at bedtime (cries,refuses to stay in bed, etc) | □ | □ | □ |
| 11.Child is afraid of sleeping in the dark | □ | □ | □ |
| 12.Child is afraid of sleeping alone | □ | □ | □ |
| 13.Child is afraid of dying during sleep | □ | □ | □ |

**Sleep Behavior：**

Child’s usual amount of sleep each day：week days：▁▁hours and▁▁minutes

（combining nighttime sleep and naps） weekends：▁▁hours and▁▁minutes

|  | Usually  5-7times/week | Sometimes  2-4times/week | | Rare/no  0-1times/week |
| --- | --- | --- | --- | --- |
| 14.Child sleeps too little‑ | □ | □ | | □ |
| 15.Child sleeps too much‑ | □ | □ | | □ |
| 16.Child sleeps the right amount each day | □ | □ | | □ |
| 17. Child's sleep quality is good | □ | □ | | □ |
| 18.Child sleeps about the same amount each day | □ | □ | | □ |
| 19.Chid wets the bed at night | □ | □ | | □ |
| 20.Child talks during sleep | □ | □ | | □ |
| 21.Child is restless and moves a lot during sleep | □ | □ | | □ |
|  | Usually  5-7times/week | Sometimes  2-4times/week | | Rare/no  0-1times/week |
| 22.Child sleepwalks during the night | □ | □ | | □ |
| 23. Children with their parents (or raiser) to sleep in the same room? | □ | □ | | □ |
| 24. Children with their parents (or raiser)sleep in the same bed | □ | □ | | □ |
| 25.Child moves to someone else’bed duringthenight(parent,brother,sister,etc) | □ | □ | | □ |
| 26.Child reports body pains during sleep. | □ | □ | | □ |
| If so,where?：▁▁▁▁▁▁ |  |  | |  |
| 27.Child grinds teeth during sleep (your dentist may have told you this) | □ | □ | | □ |
| 28.Child snores loudly | □ | □ | | □ |
| 29.Child seems to stop breathing during sleep | □ | □ | | □ |
| 30.Child snorts and/or gasps during sleep | □ | □ | | □ |
| 31.Child has trouble sleeping away from home(visiting relatives,vacation) | □ | □ | | □ |
| 32.Child compiains about problems sleeping | □ | □ | | □ |
| 33.Child awakens during night screaming,sweating,and inconsolable | □ | □ | | □ |
| 34.Child awakensalarmed by a frightening dream | □ | □ | | □ |
| **Waking During the Night：** | | | | |
|  | Usually  5-7times/week | Sometimes  2-4times/week | Rare/no  0-1times/week | |
| 35.Child awakes once during the night | □ | □ | □ | |
| 36.Child awakes more than once during the night | □ | □ | □ | |
| 37.Child returns to sleep without help after waking | □ | □ | □ | |
| 38.Write in the number of minutes a night that waking usuallylasts：▁▁hours▁▁minutes | | | | |

**Moring Waking：**

Write in the time of day chid usually wakes in the morning：weekdays：▁▁hours▁▁minutes

**weekends：▁▁hours▁▁minutes**

|  | Usually  5-7times/week | Sometimes  2-4times/week | Rare/no  0-1times/week |
| --- | --- | --- | --- |
| 39.Child wakes up by him/herself | □ | □ | □ |
| 40.Child wakes up withalarm clock | □ | □ | □ |
| 41.Child wakes up in negative mood | □ | □ | □ |
| 42.Adults or siblings wake up child | □ | □ | □ |
| 43.Ahild has difficulty getting out of bed in the moring | □ | □ | □ |
| 44.Ahild takes a long time to become alert in the moring | □ | □ | □ |
|  | Usually  5-7times/week | Sometimes  2-4times/week | Rare/no  0-1times/week |
| 45.Child wakes up very early in the moring | □ | □ | □ |
| 46.Child has a good appetite in the morning | □ | □ | □ |

Daytime Sleepiness：

|  | Usually  5-7times/week | Sometimes  2-4times/week | Rare/no  0-1times/week |
| --- | --- | --- | --- |
| 47.Child naps during the day | □ | □ | □ |
| 48.Child suddenly falls asleep in the middle of active behavior | □ | □ | □ |
| 49.Child seems tired | □ | □ | □ |

50.During the past week your child has appeared sleepy or fallen asleep during the following(check all that apply)

|  | Not sleepy | Very Sleepy | Falls Asleep |
| --- | --- | --- | --- |
| Dressing | □ | □ | □ |
| Playing alone | □ | □ | □ |
| Playing with others | □ | □ | □ |
| Watching tv | □ | □ | □ |
| Rriding in a car | □ | □ | □ |
| Eating meals | □ | □ | □ |
| Going to the bathroom | □ | □ | □ |
