# Supplementary material for: Frequent nocturnal awakening in children: prevalence, risk factors, and associations with subjective sleep perception and daytime sleepiness
Source: BMC Psychiatry. 2014 Jul 30;14:204. doi: 10.1186/1471-244X-14-204 (PMC4261897; doi:10.1186/1471-244X-14-204)
Supplement: Supplementary file 2 — Additional file 2: Table S2: The Children’s Psychosocial Screening Scale. (DOC 54 KB) [file 12888_2013_1708_MOESM2_ESM.doc]

Supplemental S2.

The Children’s Psychosocial Screening Scale

|  | Rarely | Occasionally | Sometimes | Often | Always |
| --- | --- | --- | --- | --- | --- |
| Mental and emotional condition |  |  |  |  |  |
| 1. Do you feel happy？ | ○ | ○ | ○ | ○ | ○ |
| 1. Do you often worry about something？ | ○ | ○ | ○ | ○ | ○ |
| 1. Do you often feel uneasy or nervous？ | ○ | ○ | ○ | ○ | ○ |
| 1. Do you often feel lonely or bored? | ○ | ○ | ○ | ○ | ○ |
| 1. Do you satisfied with your situation? | ○ | ○ | ○ | ○ | ○ |
| Peer acceptance and social function |  |  |  |  |  |
| 1. Do you often stay or play with your fellows/classmates? | ○ | ○ | ○ | ○ | ○ |
| 1. Do you have a lot of good friends？ | ○ | ○ | ○ | ○ | ○ |
| 1. Can you usually be understood？ | ○ | ○ | ○ | ○ | ○ |
| 1. Do you often help others？ | ○ | ○ | ○ | ○ | ○ |
| 1. Generally, do you think that most people like you? | ○ | ○ | ○ | ○ | ○ |
| Parenthood |  |  |  |  |  |
| 1. Do you love your parent(s)？ | ○ | ○ | ○ | ○ | ○ |
| 1. Do you think that your parents love you? | ○ | ○ | ○ | ○ | ○ |
| 1. Do you enjoy the time with parent(s)? | ○ | ○ | ○ | ○ | ○ |
| 1. Do you usually share your trouble with parents? | ○ | ○ | ○ | ○ | ○ |
