# Supplementary material for: Frequent nocturnal awakening in children: prevalence, risk factors, and associations with subjective sleep perception and daytime sleepiness
Source: BMC Psychiatry. 2014 Jul 30;14:204. doi: 10.1186/1471-244X-14-204 (PMC4261897; doi:10.1186/1471-244X-14-204)
Supplement: Supplementary file 3 — Additional file 3: Table S3: Sociodemographic factors regarding FNA by multivariate logistical regression models (N = 20,505). (DOC 66 KB) [file 12888_2013_1708_MOESM3_ESM.doc]

**Supplemental S3.** Sociodemographic factors regarding FNA by multivariate logistical regression models (N = 20,505)

| Variables | **Model I** | |  | **Model II** | |  | **Model III** | |  | **Model IV** | |
| --- | --- | --- | --- | --- | --- | --- | --- | --- | --- | --- | --- |
|  | Adjusted OR  (95% CI) | *P* value |  | Adjusted OR  (95% CI) | *P* value |  | Adjusted OR  (95% CI) | *P* value |  | Adjusted OR  (95% CI) | *P* value |
| **Demographic characteristics** |  |  |  |  |  |  |  |  |  |  |  |
| Age (years) |  | .082 |  |  | .028 |  |  | .184 |  |  | .211 |
| 7- vs. 5-6 | 1.18 (0.97-1.42) | .101 |  | 1.20 (0.98-1.46) | .076 |  | 1.19 (0.98-1.47) | .081 |  | 1.18 (0.97-1.46) | .080 |
| 8- vs. 5-6 | 1.15 (0.95-1.39) | .148 |  | 1.19 (0.97-1.45) | .089 |  | 1.15 (0.94-1.41) | .174 |  | 1.14 (0.94-1.40) | .261 |
| 9- vs. 5-6 | 1.27 (1.05-1.53) | .013 |  | 1.29 (1.06-1.58) | .010 |  | 1.24 (1.01-1.52) | .036 |  | 1.24 (1.01-1.52) | .042 |
| 10- vs. 5-6 | 1.26 (1.04-1.52) | .018 |  | 1.32 (1.09-1.61) | .006 |  | 1.26 (1.03-1.55) | .027 |  | 1.26 (1.01-1.54) | .041 |
| 11- vs. 5-6 | 1.31 (1.08-1.61) | .006 |  | 1.41 (1.14-1.73) | .001 |  | 1.32 (1.06-1.63) | .012 |  | 1.30 (1.05-1.61) | .021 |
| Gender (%) |  |  |  |  |  |  |  |  |  |  |  |
| Boys vs. Girl | 1.10 (1.00-1.21) | .057 |  | 1.09 (0.99-1.21) | .094 |  | 1.05 (0.95-1.68) | .345 |  | 1.06 (0.94-1.16) | .289 |
| Ethnicity | 1.26 (1.02-1.55) | .030 |  | 1.25 (1.01-1.55) | .040 |  | 1.24 (1.00-1.55) | .055 |  | 1.23 (0.99-1.54) | .053 |
| Han ethnic vs. minority ethnic |  |  |  |  |  |  |  |  |  |  |  |
| **Socioeconomic characteristics** |  |  |  |  |  |  |  |  |  |  |  |
| Family income |  | <.001 |  |  | <.001 |  |  | <.001 |  |  | <.001 |
| <800 vs. ≥2500 | 1.42 (1.20-1.69) | <.001 |  | 1.44 (1.21-1.72) | <.001 |  | 1.42 (1.19-1.70) | <.001 |  | 1.40 (1.17-1.68) | <.001 |
| 800-2500 vs. ≥2500 | 1.14 (0.99-1.32) | .066 |  | 1.16 (1.00-1.34) | .046 |  | 1.15 (1.00-1.34) | .058 |  | 1.15 (1.00-1.33) | .081 |
| Family structure |  | .001 |  |  | .001 |  |  | .004 |  |  | .007 |
| Single parent family vs. nuclear family | 1.17 (0.96-1.42) | .132 |  | 1.11 (0.90-1.36) | .333 |  | 1.01 (0.82-1.26) | .899 |  | 1.01 (0.82-1.26) | .885 |
| Large family vs. nuclear family | 0.84 (0.75-0.94) | .002 |  | 0.82 (0.74-0.92) | .001 |  | 0.82 (0.73-0.93) | .001 |  | 0.83 (0.74-0.94) | .001 |
| Mather’s education level |  | <.001 |  |  | <.001 |  |  | <.001 |  |  | <.001 |
| Low vs. high | 1.87 (1.57-2.23) | <.001 |  | 1.86 (1.55-2.23) | <.001 |  | 1.88 (1.56-2.26) | <.001 |  | 1.86 (1.55-2.27) | <.001 |
| Medium vs. high | 1.30 (1.11-1.51) | .001 |  | 1.32 (1.13-1.55) | <.001 |  | 1.31 (1.12-1.54) | .001 |  | 1.30 (1.11-1.53) | .001 |
| Mather’s education level |  | <.001 |  |  | <.001 |  |  | <.001 |  |  | <.001 |
| Low vs. high | 1.62 (1.36-1.93) | <.001 |  | 1.52 (1.27-1.82) | <.001 |  | 1.46 (1.22-1.76) | <.001 |  | 1.47 (1.22-1.76) | <.001 |
| Medium vs. high | 1.56 (1.00-1.34) | .052 |  | 1.13 (0.97-1.31) | .117 |  | 1.12 (0.96-1.31) | .152 |  | 1.13 (0.97-1.32) | .163 |

Model I adjusted for demographic and socioeconomic characteristics;

Model II adjusted for demographic and socioeconomic characteristics, sleep environments, and bedtime hygiene;

Model III adjusted for demographic and socioeconomic characteristics, sleep environments, bedtime hygiene, biological health problems, and psychosocial conditions;

Model IV adjusted for demographic and socioeconomic characteristics, sleep environments, bedtime hygiene, biological health problems, psychosocial conditions, and family history simultaneously.
